# Expression and Roles of Lynx1, a Modulator of Cholinergic Transmission, in Skeletal Muscles and Neuromuscular Junctions in Mice

**DOI:** 10.3389/fcell.2022.838612

**Published:** 2022-03-16

**Authors:** Sydney V. Doss, Sébastien Barbat-Artigas, Mikayla Lopes, Bhola Shankar Pradhan, Tomasz J. Prószyński, Richard Robitaille, Gregorio Valdez

**Affiliations:** ^1^ Department of Molecular Biology, Cellular Biology, and Biochemistry, Brown University, Providence, RI, United States; ^2^ Département de Neurosciences, Université de Montréal, Montréal, QC, Canada; ^3^ Laboratory of Synaptogenesis, Nencki Institute of Experimental Biology, Polish Academy of Sciences, Warsaw, Poland; ^4^ Laboratory of Synaptogenesis, Łukasiewicz Research Network—PORT Polish Center for Technology Development, Wrocław, Poland; ^5^ Centre de Recherche Interdisciplinaire sur le Cerveau et L’Apprentissage (CIRCA), Montreal, QC, Canada; ^6^ Center for Translational Neuroscience, Robert J. and Nancy D. Carney Institute for Brain Science and Brown Institute for Translational Science, Brown University, Providence, RI, United States; ^7^ Department of Neurology, Warren Alpert Medical School of Brown University, Providence, RI, United States

**Keywords:** synaptic plasticity, aging, acetylcholine receptor, skeletal muscle, cholinergic transmission, neuromuscular junction, Lynx1

## Abstract

Lynx1 is a glycosylphosphatidylinositol (GPI)-linked protein shown to affect synaptic plasticity through modulation of nicotinic acetylcholine receptor (nAChR) subtypes in the brain. Because of this function and structural similarity to α-bungarotoxin, which binds muscle-specific nAChRs with high affinity, Lynx1 is a promising candidate for modulating nAChRs in skeletal muscles. However, little is known about the expression and roles of Lynx1 in skeletal muscles and neuromuscular junctions (NMJs). Here, we show that Lynx1 is expressed in skeletal muscles, increases during development, and concentrates at NMJs. We also demonstrate that Lynx1 interacts with muscle-specific nAChR subunits. Additionally, we present data indicating that Lynx1 deletion alters the response of skeletal muscles to cholinergic transmission and their contractile properties. Based on these findings, we asked if Lynx1 deletion affects developing and adult NMJs. Loss of Lynx1 had no effect on NMJs at postnatal day 9 (P9) and moderately increased their size at P21. Thus, Lynx1 plays a minor role in the structural development of NMJs. In 7- and 12-month-old mice lacking Lynx1, there is a marked increase in the incidence of NMJs with age- and disease-associated morphological alterations. The loss of Lynx1 also reduced the size of adult muscle fibers. Despite these effects, Lynx1 deletion did not alter the rate of NMJ reinnervation and stability following motor axon injury. These findings suggest that Lynx1 is not required during fast remodeling of the NMJ, as is the case during reformation following crushing of motor axons and development. Instead, these data indicate that the primary role of Lynx1 may be to maintain the structure and function of adult and aging NMJs.

## Introduction

Lynx1 is a small, glycosylphosphatidylinositol (GPI)-anchored, protein shown to interact with specific types of nicotinic acetylcholine receptors (nAChRs) in the brain (Miwa et al., 1999; Ibanez-Tallon et al., 2002). Lynx1 modulates postsynaptic sensitivity *via* direct interactions with nAChRs at the postsynapse (Lyukmanova et al., 2013) and by promoting synaptic translocation of nAChR pentamers less sensitive to acetylcholine (ACh) (Nichols et al., 2014; George et al., 2017). Through this interaction with nAChRs, Lynx1 has been shown to modulate synaptic plasticity (Morishita et al., 2010; Nabel and Morishita, 2013; Takesian et al., 2018; Shenkarev et al., 2020) and has been implicated in motor learning (Miwa and Walz, 2012), nicotine addiction (Parker et al., 2017), nociception (Nissen et al., 2018), neuronal survival (Miwa et al., 2006), aging (Kobayashi et al., 2014), and Alzheimer’s disease-related pathology (Thomsen et al., 2016). Given its central role in cholinergic synaptic plasticity in the brain, and its structural similarity to α-bungarotoxin, which binds muscle-specific nAChRs with high affinity (Fleming et al., 1993), Lynx1 is a promising candidate for modulating cholinergic transmission at the neuromuscular junction (NMJ) in skeletal muscles.

As a cholinergic synapse through which motor neurons communicate with skeletal muscles, the NMJ is necessary for the initiation of all voluntary movements in mammals. Given the critical importance of the NMJ for initiating movements and survival of muscle fibers, it is not surprising that skeletal muscles have evolved a variety of mechanisms to tightly control the formation and function of NMJs. On the postsynapse, the muscle fiber region abutting the motor axon nerve ending, muscle-specific nAChR pentamers (α1, β1, δ, and γ or ε) undergo a number of changes required for the structural and functional maturation and stability of NMJs. These NMJ-associated nAChR pentamers change in number and distribution during expansion of the postsynapse (Balice-Gordon and Lichtman, 1990; Darabid et al., 2014). Their subunit composition also shifts as epsilon replaces the gamma subunit during maturation of the NMJ. This shift reduces the sensitivity of NMJ-associated nAChR pentamers to ACh (Hall and Sanes, 1993). Although nAChR pentamers are relatively stable in healthy adult NMJs, this is not the case at NMJs affected by diseases and injuries. These conditions increase the expression of the gamma subunit in addition to phosphorylation and trafficking of nAChRs (Smith et al., 1990; Misgeld et al., 2002, 2005; Leenders and Sheng, 2005; Friese et al., 2007; Lanuza et al., 2010). These changes in nAChRs are critical for the proper development, stability, and function of NMJs and muscle fibers (Sanes and Lichtman, 1999). Because of its regulation of CNS nAChRs (Lyukmanova et al., 2013), it is possible that skeletal muscles use Lynx1 to regulate NMJ-associated nAChRs.

In this study, we deployed molecular, cellular, genetic, and electrophysiological techniques to examine Lynx1 in skeletal muscles and NMJs of developing and adult mice. We show that Lynx1 is present in skeletal muscles and interacts with muscle-specific nAChRs. We demonstrate that Lynx1 influences the response of muscles to cholinergic transmission and their contractile properties. We also show that the primary role of Lynx1 is to maintain the stability of adult NMJs.

## Materials and Methods

### Animals

Lynx1^−/−^ mice (Miwa et al., 2006) were obtained from the lab of Dr. Morishita and mated with Thy1-YFP16 (Feng et al., 2000) (RRID:IMSR_JAX:003,709) animals to generate Lynx1^−/−^;Thy1-YFP16 animals. Heterozygous vesicular acetylcholine transporter (VAChT) knockdown mice (Prado et al., 2006) were generously donated by Dr. Marco Prado. Male and female mice were used in this study. Mice were sacrificed with a lethal dose of isoflurane. According to the needs of the experiments, muscles were immediately dissected for collection of fresh frozen tissues; transcardial perfusion with 1X phosphate-buffered saline (PBS) (pH 7.4) followed by 4% paraformaldehyde (PFA, pH 7.4) was performed to fix tissues, or tissues were collected in a physiological solution for electrophysiology. All experiments were carried out under the NIH and Canadian Council of Animal Care guidelines and animal protocols approved by the Brown University Institutional Animal Care and Use Committee (IACUC #19-05-0013) and the Comité de déontologie animale of Université de Montréal.

### C2C12 Cultures

C2C12 cells were plated in eight-well Flexiperm chambers on Permanox slides (Thermo Fisher, 160005) coated with poly-L-ornithine (3 μg/ml; Sigma-Aldrich; P2533) and laminin (10 μg/ml; Thermo Fisher Scientific; 23017015) in Dulbecco’s Modified Eagle Medium (DMEM, Life Technologies). Myoblasts were plated at 100,000 cells per well in culture media (high-glucose DMEM, 20% fetal bovine serum, 1× glutamine, pen–strep, and Fungizone) and incubated at 37°C and 5.0% CO_2_. Twenty-four hours post-plating, the media were replaced with fusion media (high-glucose DMEM, 2% horse serum, 1× glutamine, pen–strep, and Fungizone). Myoblasts were then incubated for 3–7 days following the addition of fusion media to generate myotubes. C2C12 myotubes were treated for 24 h with recombinant z-agrin (10 ng/ml) or 10 µM carbachol (CCH). RNA was extracted from myotubes at 0, 3, and 7 days post-fusion using an Aurum Total RNA mini kit (Bio-Rad), following manufacturer’s instructions.

### Affinity Pull-Down Assays

#### Plasmid Constructs

The coding sequence of the α1 nAChR subunit ectodomain (amino acids 21–230), with the signal sequences removed, was cloned into a pGEX-4T1 vector. The coding sequence of Lynx1 (amino acids 21–92) with the signal sequence and the GPI anchoring signal removed (Lyukmanova et al., 2011) was cloned into the pGEX-4T1 vector. The plasmids encoding the full-length α1, β1, δ, and ε nAChR subunits were a kind gift from Dr. Lin Mei. The plasmid pLynx1-mCherry, as described in Nichols et al. (2014), was obtained from Dr. Henry A. Lester.

#### Recombinant Protein Purification

The glutathione S-transferase (GST)-tagged nAChR α1 subunit ectodomain and Lynx1 were purified as previously described (Herrmann et al., 2015). BL21 *E. coli* were transformed with the respective constructs, grown in 200-ml cultures at 18°C until achieving a 600-nm optical density between 0.4 and 0.6. Protein expression was induced by 0.5 mM isopropyl 1-thio-β-d-galactopyranoside (IPTG) for 4 h at 18°C. Bacteria were collected, incubated for 30 min at 4°C in lysis buffer (50 mM NaH_2_PO_4_, 300 mM NaCl, 10 μg/ml leupeptin, 10 μg/ml aprotinin, 1 μl/ml Triton X-100, 10 μg/ml DNase I, and 15 units/μl lysozyme), lysed by sonication for 1 min, and centrifuged at 13.8×*g* for 30 min. The supernatants containing the GST fusion proteins were incubated overnight with 20 μl of equilibrated glutathione beads (GE Healthcare #17-0756-01). The beads were washed three times with washing buffer (4.3 mM Na_2_HPO_4_, 1.47 mM KH_2_PO_4_, 137 mM NaCl, and 2.7 mM KCl), and the proteins were eluted with elution buffer (10 mM glutathione, 50 mM Tris, pH 8.0).

#### nAChR Co-Immunoprecipitation

HEK293 cells (1 × 10^6^ cells/transfection) were co-transfected with pLynx1-mCherry and four nAChR subunits (α1, β1, δ, and ε). At 36 h post-transfection, cell lysates were obtained following addition of ice-cold lysis buffer (50 mM Tris-HCl, 150 mM NaCl, 50 mM NaH_2_PO_4_, 10 mM imidazole, 0.1% NP40, 10% glycerol, protease inhibitor cocktail, and 50 mM DTT, pH 8.0), incubation on a rotary shaker (1 h, 4°C), and centrifugation at 13,000×*g* at 4°C. The immunoprecipitation was performed as previously described (Gingras et al., 2016). Briefly, the lysates were centrifuged, and supernatants were incubated with Dynabeads (Invitrogen, #10003D) and coated with anti-AChR antibody (BioLegend, #838301). Beads were then washed three times with lysis buffer, resuspended in 2× sample buffer (4% SDS, 20% glycerol, 200 mM DTT, and 0.01% bromophenol blue), and boiled for 5 min. The samples were resolved by SDS-PAGE and analyzed with anti-AChR antibody (BioLegend, #838301) and anti-red fluorescent protein (RFP) antibody (Rockland, #600-401-379), which cross-reacts with mCherry and other RFP variants.

To determine the direct interaction of GST-Lynx1 with the extracellular domain of the nAChR α1 subunit, the truncated GST-tagged nAChR α1 ectodomain was incubated with bungarotoxin-biotin-coated (Thermo Fisher Scientific, #B1196) Streptavidin Dynabeads (Thermo Fisher Scientific, # 11205D) for 1 h at 4°C. Beads were then washed three times with lysis buffer and incubated with the purified GST-Lynx1 overnight. Beads were then washed three times with lysis buffer, resuspended in 2X sample buffer, and boiled for 5 min. The samples were resolved by SDS-PAGE and analyzed with anti-GST antibody.

### Lynx1 Antibody Generation

An antibody against Lynx1 was developed following the methods previously outlined (Goodman et al., 2016). In brief, a piggyback transposon vector pXL-CAG-zeomycin-2A and a piggyback transposase vector pCAG-mPBorf were obtained as a gift from Dr. Joshua Sanes. The Lynx1 sequence was cloned into the transposon vector following the 2A peptide sequence. L-cells were co-transfected with pXL-CAG-zeomycin-2A-Lynx1 and pCAG-mPBorf. A stable cell line of Lynx1-expressing L-cells was generated by selection with zeomycin, and expression analysis confirmed the presence of Lynx1 mRNA. An antibody against mouse Lynx1 was generated by immunizing 1-month-old Lynx1^−/−^ mice with Lynx1-expressing L cells. Total serum was collected from immunized mice after 6 weeks of immunizations and purified using acetone powder. Antibody specificity was verified using transfected L cells and knockout mouse tissue.

### Electrophysiology Recordings

#### Nerve-Muscle Preparations

Nerve-muscle preparations of the extensor digitorum longus (EDL) muscle were dissected from 4-month-old males in oxygenated physiological solution (in millimolar): 110 NaCl, 5 KCl, 1 MgCl_2_, 25 NaHCO_3_, 2 CaCl_2_, 11 glucose, 0.3 glutamic acid, 0.4 glutamine, 5 BES [N,N-Bis(2-hydroxyethyl)-2-aminoethanesulfonic acid sodium salt], 0.036 choline chloride, and 4.34 × 10^−7^ cocarboxylase. After dissection, nerve-muscle preparations were pinned in a SYLGARD-coated recording chamber constantly perfused with oxygenated physiological solution (95% O_2_ and 5% CO_2_). The pH (7.4) and temperature (28 ± 2°C) were continuously regulated.

#### Recordings of Synaptic Transmission

Only recordings with an initial membrane potential more negative than −65 mV and with less than 5 mV variation from holding potential were included in the analysis. NMJs were located using bright field illumination of an upright Olympus microscope with a ×60 objective. Muscle fibers were impaled 50–100 µm from the NMJ to be studied, avoiding mechanical distortion of the NMJ, but close enough to minimize the impact of spatial attenuation owing to the long muscle fiber space constant (400–1,000 µm) (Katz and Miledi, 1968; Tremblay et al., 1985). This is supported by the data showing small variability in MEPP rise time and slope (Figure 3).

Stimulation of the deep peroneal nerve was performed using a suction electrode filled with extracellular saline. Endplate potentials (EPPs) were recorded using glass microelectrodes (1.0 mm OD; WPI) pulled to 40–70 MΩ (filled with 3 mM KCl) with a P-70 Brown-Flaming micropipette puller (Sutter Instruments). Synaptic responses were amplified by an AM Systems 1,600 amplifier and further amplified (×100) and filtered (2 kHz) by a Warner Instruments DC amplifier. The recordings were digitized (10 kHz) using a National Instruments BNC 2110 board and subsequently acquired with WinWCP software (John Dempster, Strathclyde University, Strathclyde, United Kingdom).

Synaptic strength was determined by measuring the paired pulse facilitation (PPF) and the quantal content (m). These were obtained using a low-Ca^2+^ (1 mM) and high-Mg^2+^ (7.0 mM) modified physiological solution. Miniature endplate potential (MEPP) amplitude and frequency were first determined during a 5–10-min period of recordings without motor nerve stimulation. PPF was then obtained using two stimuli (0.1-m duration and 10-m interval), elicited at 0.2 Hz. Quantal content (m) was determined using the amplitude of the first EPP (EPP1) and MEPPs (mean EPP1s amp / mean MEPPs amp). Four to seven NMJs were studied per muscle.

Following a baseline recording of 20 min (0.2 Hz), synaptic plasticity was elicited by a sustained motor nerve stimulation (120 Hz, 10 s) followed by 45-min recordings of EPPs evoked at 0.2 Hz. Muscle contractions were prevented with partial blockade of the postsynaptic ACh receptors using D-tubocurarine (2.0 µM, Sigma). Only one NMJ was studied per muscle.

#### Muscular Strength and Fatigue

EDL nerve-muscle preparations were attached to a fixed force transducer (model 402A-500 mN, Aurora Scientific Inc.) at one end and an adjustable hook at the other end, using surgical thread. The knots for attaching the muscle to the force transducer and the hook were done at the level of the tendons, under a binocular, to prevent muscle fiber damage. Muscles were maintained vertically in a 140-ml beaker containing oxygenated physiological solution. Two platinum wires were positioned on the muscle and on the tendon to stimulate the muscle directly.

Muscular twitch force responses were elicited by means of single supra-maximal square-wave pulses lasting 1 and 0.1 ms, respectively. Optimal muscle length was determined by incrementally stretching the muscle until maximum neuro-muscular twitch force output was attained. After each length adjustment, a 2-min rest period was allowed before the next stimulation.

The fatigue protocol consisted of bouts of motor nerve stimulations (120 Hz for 300 ms) at 1 Hz, for 3 min. Muscular stimulations were combined with nerve stimulations (data not shown) at the 2nd and 10th stimulation and every 10th stimulation thereafter until the end of the fatigue protocol. This was followed by a 30-min recovery period where muscular and strength was measured at 2, 5, 10, 15, and 30 s and 1, 1.5, 2, 2.5, 5, 10, 20, and 30 min following the fatigue protocol.

### Fibular Nerve Crush

A fibular nerve crush was performed on 3-month-old Lynx1^−/−^;Thy1YFP16 and Thy1-YFP16 control mice, as described previously (Dalkin et al., 2016). In brief, the fibular nerve was crushed at its intersection with the lateral head of the gastrocnemius muscle tendon, near the knee. This technique creates a highly reproducible injury with a well-characterized pattern of degeneration and re-innervation in wild-type mice. In young adult mice, nAChRs become completely denervated by 4 days post-injury, motor axons begin to contact the post-synapse around 7 days, and the NMJ is fully innervated by 12 days post-injury.

### Immunohistochemistry and Confocal Microscopy of Extensor Digitorum Longus Muscles

EDL muscles from Thy1-YFP16 mice expressing YFP in nerve endings were used to visualize NMJs. Following perfusion, muscles were dissected and incubated with Alexa Fluor 555-conjugated α-bungarotoxin (555-fBTX, Invitrogen, #B35451, 1:1,000 in 1× PBS) for 1 h. Muscles were then washed three times with 1× PBS and whole mounted in VECTASHIELD (Vector Laboratories). For Lynx1 IHC, EDL muscles were incubated for 1 h at room temperature in blocking buffer (1× PBS, 5% bovine serum albumin, 3% goat serum, 0.5% Triton-X), incubated overnight at 4°C in Lynx1 antibody diluted 1:10 in blocking buffer, washed three times with 1× PBS, incubated for 2 h at room temperature in Alexa Fluor 488-conjugated polyclonal anti-mouse IgG antibody (Invitrogen # A-11001, 1:1,000) and 555-fBTX (1:1,000) diluted in blocking buffer, washed three times with 1× PBS, and whole mounted in VECTASHIELD. NMJs were imaged using a Zeiss LSM 700 confocal microscope. Maximum intensity projections from confocal z-stacks were created using Zen Black software (Zeiss).

#### NMJ Analysis

Structural features were analyzed based on previously described methods (Valdez et al., 2010). In brief, a minimum of 50 NMJs in the EDL muscle were randomly sampled using the grid function in ImageJ. Full or partial denervation of NMJs was classified by incomplete apposition of a YFP-labeled motor axon terminal with fBTX-labeled nAChR clusters. Fragmentation was determined by counting the number of discrete fBTX-labeled nAChR islands per NMJ. Sprouting NMJs were those with a nerve terminal overreaching the nAChR cluster. NMJs with multiple innervations were those with more than one axon innervating a single nAChR cluster. Terminal blebs were defined as a bulbous motor axon terminal whose outermost edges did not directly appose the fBTX-labeled postsynapse. Axon blebs were defined as bulbous regions of the motor axon terminal located in close proximity to the NMJ. The endplate area was determined by measuring the area enclosed by the perimeter of nAChR clusters of a given NM, including fBTX^+^ and fBTX^−^ pixels. nAChR area was determined by measuring the area of fBTX^+^ pixels of a given NMJ. AChR dispersion was calculated by dividing the endplate area by the AChR area for a given NMJ. Images were blinded for analysis.

### Immunohistochemistry and Confocal Microscopy of TA Cross-Sections

PFA-fixed TA muscles were dissected and incubated in 30% sucrose for 48 h at 4°C. Muscles were then cut in half and placed in base molds (Fisherbrand) with Tissue Freezing Medium (General Data Healthcare). Using a cryostat, TA muscles were cross-sectioned at 16-µm thickness and collected on gelatin-coated slides. Sections were first washed three times with 1× PBS and then incubated for 1 h at room temperature with wheat germ agglutinin conjugated with Alexa Fluor 555 (WGA, 1:700) and DAPI (4′6-diamidino-2-phenylindole, Sigma-Aldrich; 2818-90-3; 1:1,000) diluted in 1× PBS. Muscles were then washed three times with 1× PBS and whole mounted using VECTASHIELD (Vector Laboratories). Muscle fibers were imaged using a Zeiss LSM 900 confocal microscope using a ×20 objective (0.8 numerical aperture). Maximum intensity projections from confocal z-stacks were created using Zen Black (Zeiss) and analyzed using ImageJ. Muscle fiber perimeters were identified by laminin IHC (Rabbit anti-laminin, Sigma #L9393), and the area within the perimeter was measured in ImageJ using the grid function to randomly select at least 220 fibers across four muscle sections per mouse. Muscle fibers with centralized nuclei were identified by the presence of a DAPI-labeled nucleus near its center. Images were blinded for analysis.

### Imaging

Images were obtained with a Zeiss LSM 710 or Zeiss LSM 900 laser scanning confocal microscope (Carl Zeiss Microscopy, Berlin, Germany) using a ×20 (0.8 numerical aperture) objective. Maximum intensity projections and stitching of tile scans were generated with Zeiss Zen software.

### Quantitative Polymerase Chain Reaction Expression Analysis

RNA was isolated from flash-frozen TA muscles using an Aurum Total RNA Mini Kit (Bio-Rad), following the manufacturer’s instructions. cDNA was then synthesized from 100 ng of total RNA using an iScript cDNA synthesis kit (Bio-Rad). qPCR was performed on the Bio-Rad CFX Connect Real-Time System (Bio-Rad) using iTaq Universal SYBR Green Supermix (Bio-Rad). Expression values were normalized to GAPDH using the 2^−ΔΔCT^ method. The primers used in this study are listed in Table 1.

**TABLE 1:** qPCR primer sequences.

Gene	Forward primer (5′-3′)	Reverse primer (5′-3′)
AChE	CTA​CAC​CAC​GGA​GGA​GAG​GA	CTG​GTT​CTT​CCA​GTG​CAC​CA
*Chrna1*	CTT​CAA​AGA​GCT​TTG​CCA​CC	CCA​TGG​AGC​TCT​CGA​CTG​TT
*Chrnb1*	AGG​TCT​CAG​GCA​CTT​TGT​CG	TTC​TAC​CTC​CCA​CCA​GAT​GC
*Chrnd*	CCG​ATG​CAC​TAT​CTC​CCA​CT	CTT​AGC​CTG​AAG​CAG​GAG​GA
*Chrne*	GCT​GTG​TGG​ATG​CTG​TGA​AC	GCT​GCC​CAA​AAA​CAG​ACA​TT
GAPDH	CCC​ACT​CTT​CCA​CCT​TCG​ATG	GTC​CAC​CAC​CCT​GTT​GCT​GTA​G
Lynx1	ACC​ACT​CGA​ACT​TAC​TTC​ACC	ATC​GTA​CAC​GGT​CTC​AAA​GC

### Exercise Wheel

Mice aged 5 or 9 months were housed individually in a Mouse Activity Wheel Chamber with Filter Lid (Lafayette Instruments Neuroscience; Model 80820F) with a 12-h light/dark cycle and access to food and water *ad libitum*. Their activity was monitored continuously with Activity Wheel Monitor Software (Lafayette Instruments Neuroscience; Model 86065) for 11 weeks (7 months age group) or 14 weeks (12 months age group). Four Thy1-YFP16 male mice and five Lynx1^−/−^;Thy1-YFP16 male mice were analyzed in each age group.

### Statistics

For comparisons between two experimental groups, unpaired two-sided Student’s *t*-tests or Welch’s unpaired Student’s *t*-test, based on an *F*-test of variance, were used to determine significance. Comparisons between three or more experimental groups were made with one-way ANOVA with Bonferroni multiple comparisons test, and comparisons of two independent variables were made with two-way ANOVA with Šídák’s multiple comparisons test. Comparisons of muscle fiber CSA distributions were made with a Kolmogorov–Smirnov test. *p*-values and sample sizes (*n*) are described in the figure legends. An *n* was defined as one animal for all experiments except comparisons of muscle fiber size distribution, where an *n* was defined as one muscle fiber, and Lynx1 expression in C2C12 cells, where an *n* was defined as a cell culture from an independent passage. Data are expressed as mean ± standard deviation unless otherwise noted. A *p*-value <0.05 was considered statistically significant. Statistical analysis was performed using GraphPad Prism9 and Microsoft Excel Data Analysis plugin.

## Results

### Lynx1 Concentrates in the Postsynaptic Region of the Neuromuscular Junction

**FIGURE 1:**
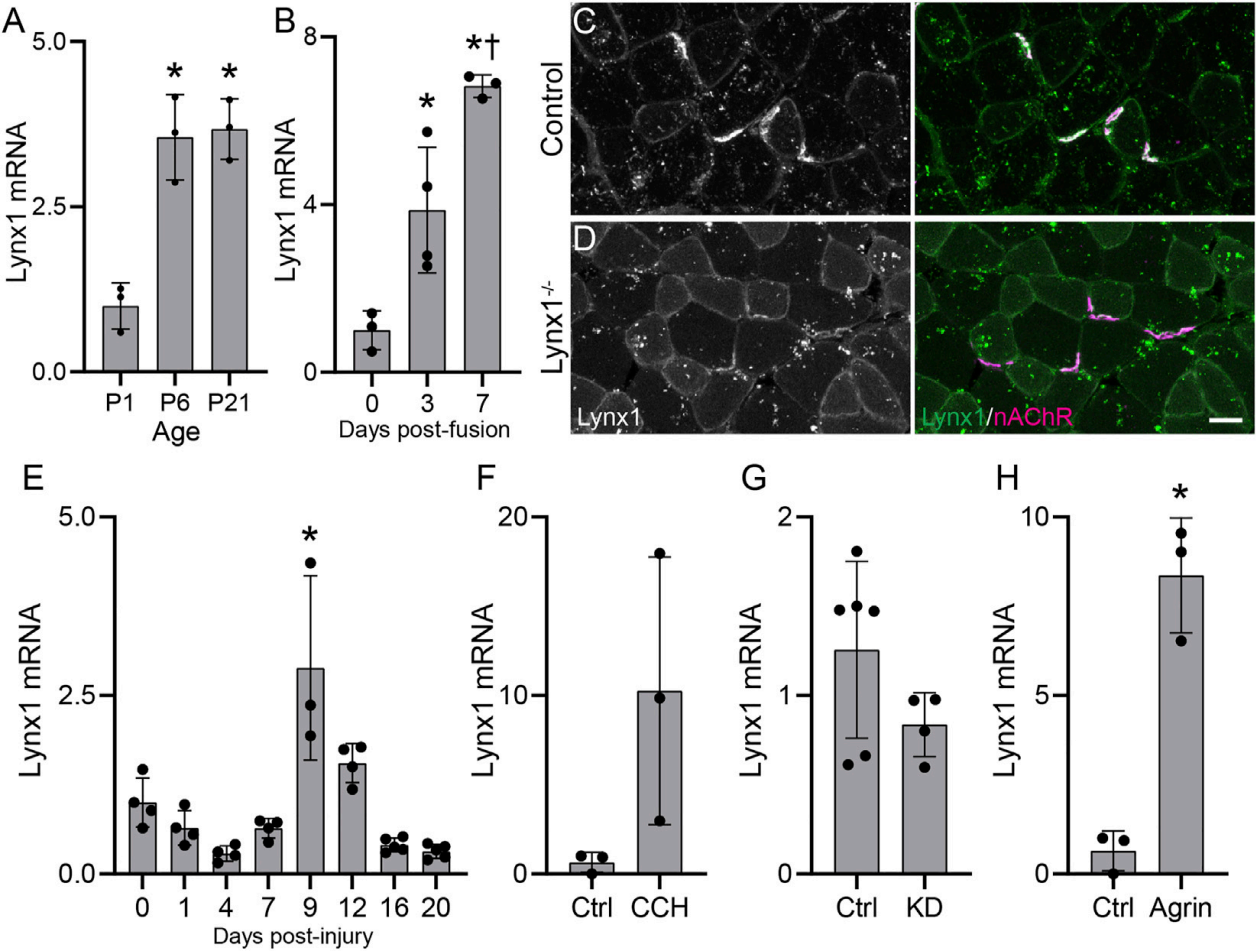
Lynx1 levels track structural and functional changes at neuromuscular junctions (NMJs). **(A,B)** qPCR analysis of Lynx1 mRNA levels in **(A)** developing tibialis anterior (TA) and extensor digitorum longus (EDL) muscles and in **(B)** C2C12 myotubes at 3 and 7 days post-fusion compared to unfused myoblasts; *p < 0.05 versus P1 or control. ^†^ *p* < 0.05 versus 3 days post-fusion, one-way ANOVA with Bonferroni *post hoc*. **(C,D)** Representative images of Lynx1 (green) and fluorescently conjugated α-bungarotoxin (fBTX)-labeled nicotinic acetylcholine receptors (magenta) in control and Lynx1^−/−^ TA muscle cross-sections. **(E,F)** qPCR analysis of Lynx1 mRNA levels in **(E)** the TA muscle following fibular nerve crush injury, **(F)** C2C12 myotubes following 24-h carbachol (CCH) treatment, **(G)** the TA muscle of vesicular acetylcholine transporter (VAChT) knockdown (KD) mice, and **(H)** C2C12 myotubes following 24-h z-agrin treatment. *p < 0.05 versus P1 or control, one-way ANOVA with Bonferroni *post hoc*. All values are mean ± SD. Scale bar = 25 µm.

We first examined the expression of Lynx1 in developing skeletal muscles of wild-type mice. We found Lynx1 transcripts expressed at higher levels at postnatal (P) day 6 and P21 compared to P1 in the TA and EDL muscles (Figure 1A). We also found higher levels of Lynx1 in more matured C2C12-derived myotubes (Figure 1B). To assess the relationship between Lynx1 and the NMJ, we generated an antibody to immunostain skeletal muscles for Lynx1. This antibody showed an enrichment of Lynx1 protein at NMJs in wild-type (WT) control muscle (Figure 1C), even though it also binds to other molecules in muscles given the signal seen around the peripheral membrane of muscles fibers in Lynx1^−/−^ mice (Figure 1D). These complementary experiments demonstrate that muscle fibers express and concentrate Lynx1 at NMJs.

### Lynx1 Levels Track Structural and Functional Changes at Neuromuscular Junctions

The temporal expression pattern and concentration at NMJs led us to ask if Lynx1 expression correlates with structural and possibly functional changes at NMJs. To better understand this relationship, we examined Lynx1 levels in adult WT mice following degeneration and regeneration of motor axons, which result in fast and dramatic structural and functional changes at NMJs. Using the fibular nerve crush paradigm (Dalkin et al., 2016), we found that Lynx1 levels indeed correlate with changes at NMJs in the EDL muscle. Lynx1 expression decreased between 1 and 7 days post-injury (Figure 1E), stages when NMJs are completely denervated and then begin to be reinnervated. Lynx1 expression then increases at 9 days post-injury (DPI) and remains elevated at 12 DPI, a time span when NMJs reacquire their normal structural characteristics. Interestingly, Lynx1 is expressed at lower levels in muscles at 16 and 20 DPI, long after NMJs have reacquired their normal cellular architecture, compared to uninjured control muscles. This finding suggests that denervation causes long-term expression and possibly functional changes in skeletal muscles. Together, these data show that Lynx1 levels correlate with the structural and functional status of NMJs.

The data above suggested that either synaptic activity, factors important for NMJ maturation and stability, or both, may influence Lynx1 expression in muscle fibers. To explore this possibility, we examined the influence of ACh and neural agrin (z-agrin), two nerve-derived molecules essential for cholinergic transmission and sculpting the postsynapse (Sanes and Lichtman, 1999), on Lynx1 expression in cultured C2C12 myotubes. In culture, C2C12 myotubes express nAChRs, which aggregate into postsynaptic-like plaques and respond to cholinergic agonists (Henning et al., 1996; Kummer et al., 2004), as well as muscarinic AChRs (Liu et al., 2002). To test the effects of ACh, we treated C2C12 myotubes with the ACh mimetic carbachol (CCH) for 24 h. We found that activation of nAChRs following CCH treatment increased Lynx1 expression in C2C12 myotubes (Figure 1F). To examine more closely the relationship between Lynx1 levels and cholinergic transmission, we examined its expression in the TA muscle of transgenic mice with reduced cholinergic tone using transgenic mice deficient for the vesicular acetylcholine transporter (VAChT^KD−Het^) (Prado et al., 2006, 2013). We found that Lynx1 was expressed at lower levels in VAChT^KD−Het^ mice (Figure 1G). Finally, we treated C2C12 myotubes with the motor axon-derived nAChR clustering molecule z-agrin. We found that, similar to CCH, z-agrin treatment induced Lynx1 expression (Figure 1H).

### Lynx1 Interacts With nAChRs of the Neuromuscular Junction

**FIGURE 2:**
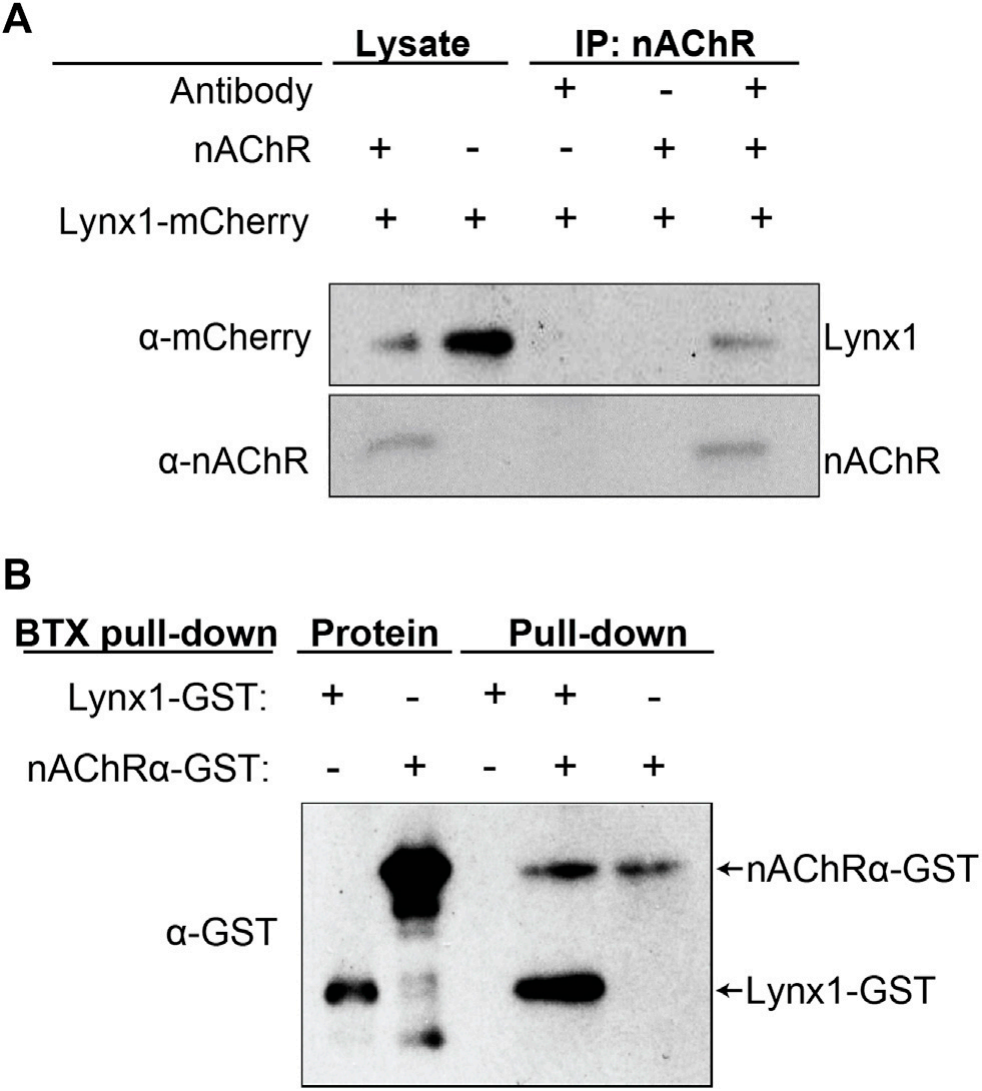
Lynx1 interacts with nicotinic acetylcholine receptors (nAChRs). **(A)** nAChR co-immunoprecipitation (co-IP) of HEK293 cells co-transfected with Lynx1-mCherry and the α1, β1, δ, and ε nAChR subunits. Western blot was performed with anti-mCherry or anti-nAChR antibodies. Lane 1, cell lysate transfected with Lynx1-mCherry and nAChR subunits. Lane 2, cell lysate transfected with Lynx1-mCherry only. Lane 3, nAChR co-IP of cell lysate transfected with Lynx1-mCherry only. Lane 4, no antibody co-IP of cell lysate transfected with Lynx1-mCherry and nAChR subunits. Lane 5, nAChR co-IP of cell lysate transfected with Lynx1-mCherry and nAChR subunits. **(B)** α-bungarotoxin (BTX) pull-down of purified recombinant glutathione S-transferase (GST)-tagged truncated α1 nAChR and GST-tagged Lynx1. Western blot was performed with a GST antibody. Lanes 1 and 2 show GST-tagged Lynx1 and GST-tagged truncated α1 nAChR at their predicted sizes. Lanes 3–5 show BTX pull-down products of samples with and without recombinant GST-tagged truncated α1 nAChR or GST-tagged Lynx1. *n* ≥ 3.

Lynx1 was found to closely associate with neuronal nAChR subunits (Miwa et al., 1999, 2006; Ibanez-Tallon et al., 2002; Lyukmanova et al., 2013; Nichols et al., 2014; George et al., 2017), with the acetylcholine-binding protein (AChBP) from *L. stagnalis*, and with muscle nAChRs in *Torpedo californica* (Lyukmanova et al., 2013). However, it remains unknown if Lynx1 interacts with mammalian NMJ-specific nAChR subunits. We performed a series of biochemical experiments to analyze the interaction of Lynx1 with an nAChR pentamer consisting of the four subunits unique to mammalian adult NMJs (α1, β1, δ, and ε). We co-transfected HEK293 cells with all four nAChR subunits along with mCherry-tagged Lynx1 (mCherry-Lynx1) (Nichols et al., 2014). Demonstrating successful co-transfection, both nAChR pentamers and mCherry-Lynx1 were readily detected in cell lysates from HEK293 cells with western blot using antibodies against the nAChR pentamer and mCherry (Figure 2A, lane 1). Specificity of the nAChR antibody was confirmed by western blot of HEK293 cell lysate collected following transfection of Lynx1-mCherry but not the nAChR subunits (Figure 2A, lane 2). We then performed co-immunoprecipitation (co-IP) assays of proteins from transfected HEK293 cell lysates using an antibody against the nAChR and probed precipitates on western blots for both nAChR and mCherry. We found that mCherry-Lynx1 co-immunoprecipitated only from HEK293 cells co-expressing nAChR pentamers (Figure 2A, lanes 3–5). This finding suggests that Lynx1 interacts with the muscle-specific nAChR pentamer located at mammalian NMJs.

We next tested the ability of α-bungarotoxin (BTX), which binds the α1 nAChR subunit with high affinity (Dellisanti et al., 2007), to pull down Lynx1 from a solution of purified recombinant GST-tagged Lynx1 and α1 nAChR ectodomain proteins. Providing evidence for a direct interaction between Lynx1 and an NMJ-specific nAChR pentamer, we found that BTX pulls down both the α1 nAChR subunit and Lynx1, as revealed by western blot using an anti-GST antibody (Figure 2B). Collectively, these experiments strongly indicate that Lynx1 directly interacts with muscle-specific nAChR pentamers found in mammalian NMJs.

### Lynx1 Modulates the Kinetics of Miniature Endplate Potentials

**FIGURE 3:**
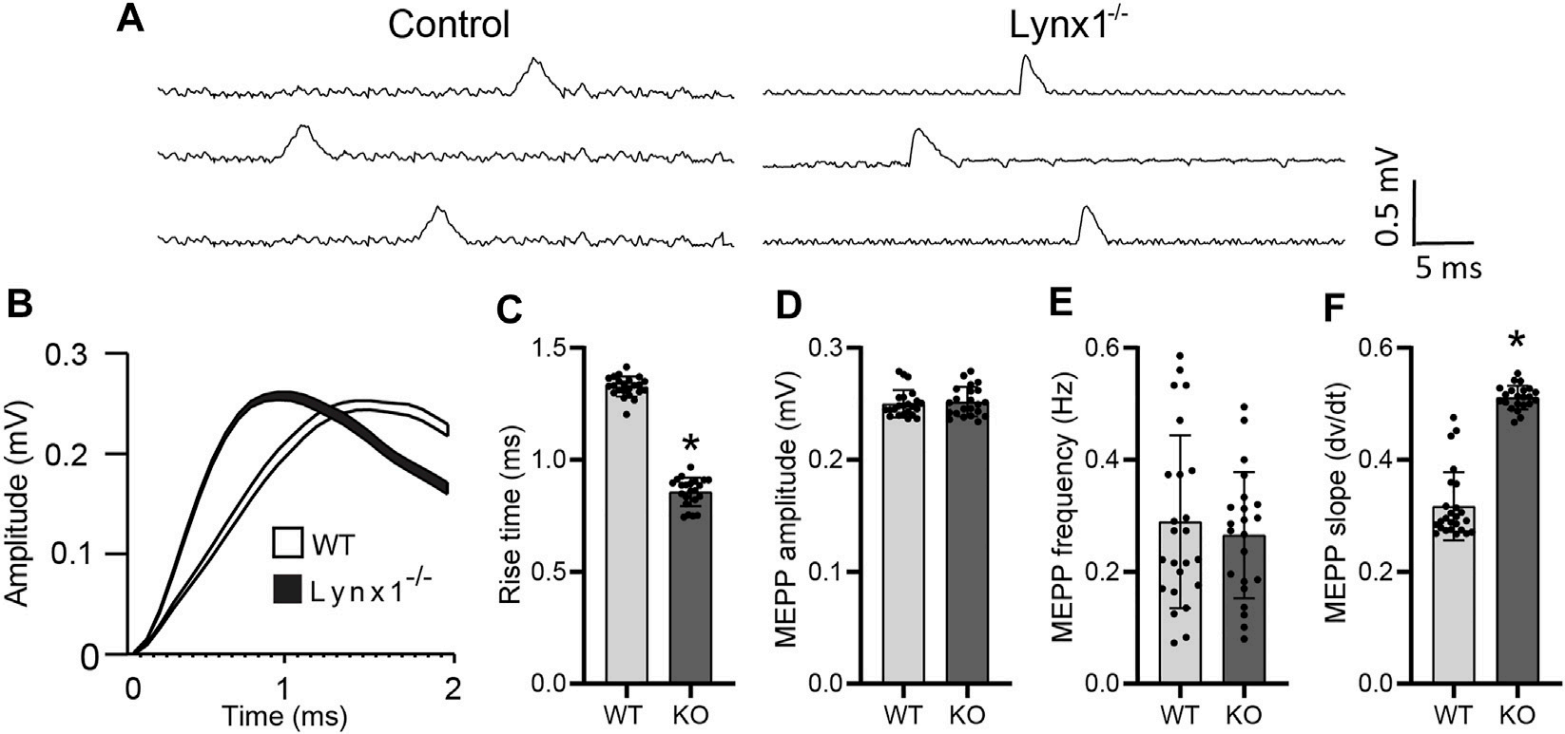
Lynx1 deletion increases nAChR sensitivity. **(A)** Example traces of spontaneous miniature endplate potential (MEPP) recordings from control and Lynx1^−/−^ extensor digitorum longus (EDL). **(B)** The average MEPP amplitude of control and Lynx1^−/−^ muscle, where the line width represents the SEM of 100 recordings. **(C)** The average rise time to peak amplitude of MEPPs represented in **(B)**. **(D)** The mean amplitude of MEPPs in control and Lynx1^−/−^ muscle. **(E)** The frequency of MEPPs in control and Lynx1^−/−^ muscle. **(F)** The average slope of MEPPs to peak amplitude in **(B)**. Control *n* ≥ 5, Lynx1^−/−^ *n* ≥ 8. All values are mean ± SD. *p < 0.05, unpaired, two-tailed Student’s *t*-test.

**FIGURE 4:**
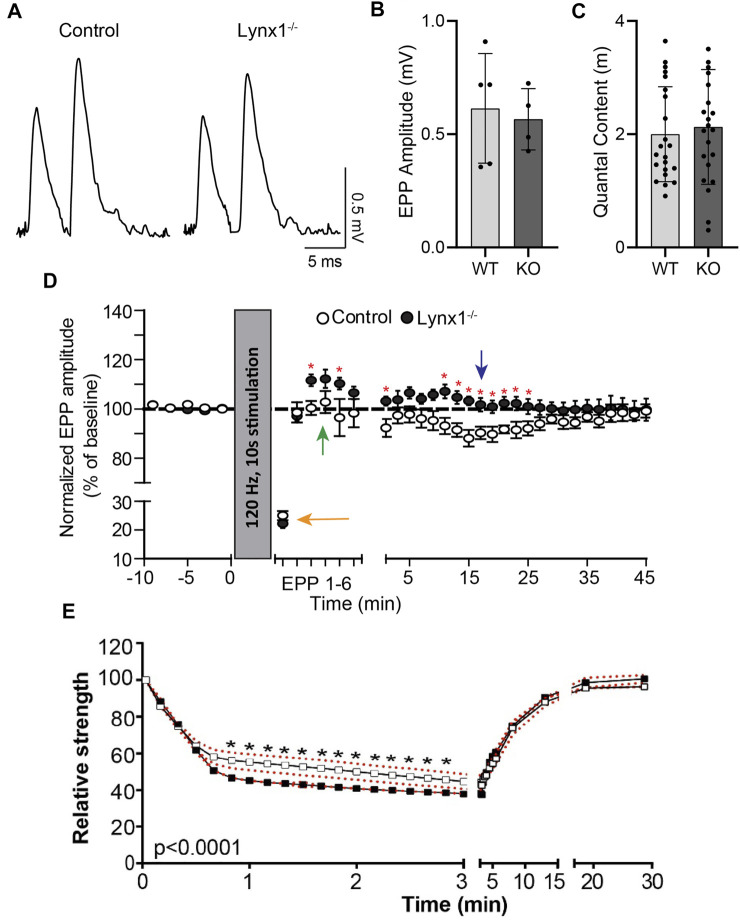
Lynx1 reduces synaptic plasticity. **(A)** Example recordings of endplate potentials (EPPs) elicited by paired-pulse stimulation (0.2 Hz, 10-m interval) from control and Lynx1^−/−^ extensor digitorum longus (EDL). **(B)** The average EPP amplitude (measured of the first EPP of the pair) and **(C)** the average quantal content following paired-pulse stimulation. **(D)** The amplitude of EPPs at baseline and following tetanic stimulation (120 Hz, 10 s). The orange arrow denotes rapid depolarization following initial stimulation. The green arrow denotes post-tetanic potentiation in Lynx1^−/−^ but not control muscle. The blue arrow denotes the absence of long-lasting depression in Lynx1^−/−^ muscle. **(E)** Neuromuscular fatigue represented as relative strength, as a percent of baseline, following super-imposed muscle stimulations after fatigue protocol in 4-month-old control and Lynx1^−/−^ EDL (red dotted line represents SEM). Values in **(B)** and **(C)** are mean ± SD, and values in **(D)** and **(E)** are mean ± SEM. *p < 0.05, unpaired, two-tailed Student’s *t*-test.

We sought to determine if Lynx1 also modulates the function of nAChRs at the NMJ. We examined the kinetics of MEPPs as a readout of the biophysical properties of postsynaptic nAChRs in NMJs of 4-month-old Lynx1^−/−^ and WT control mice. This analysis showed that the rise time was significantly faster in NMJs of Lynx1^−/−^ compared to control mice (control = 1.33 ± 0.1052 ms; Lynx1^−/−^ = 0.8576 ± 0.13 ms, *p* = 1.66 × 10^−27^; Figure 3A–C). Supporting this finding, the slope to reach the maximum MEPP amplitude was steeper at NMJs lacking Lynx1 (Figure 3F). Despite these differences, deletion of Lynx1 did not affect the mean amplitude (Figure 3D) or frequency (Figure 3E) of MEPPs. Furthermore, loss of Lynx1 did not alter the EPP amplitude (Figure 4A, B) or the quantal content (Figure 4C) at NMJs. These data suggest that loss of Lynx1 alters the biophysical properties of the postsynaptic region possibly due to changes in nAChRs.

We next examined if Lynx1 altered synaptic transmission upon repetitive stimulation. For this, we compared EPP amplitude between control and Lynx1^−/−^ muscles following sustained motor nerve stimulation (120 Hz at 10-s intervals). At NMJs of control mice, we observed a characteristic early rapid depression (74.96% ± 1.59%; Figure 4D, orange arrow) that was followed by a delayed depression (11.88% ± 3.64%; Figure 4D, blue arrow). In NMJs of Lynx1^−/−^ mice, a characteristic rapid synaptic depression following initial stimulation was produced (77.74% ± 1.55%; Figure 4D, orange arrow); however, this was followed by a significant post-tetanic potentiation event (12.22% ± 3.69%) (Figure 4D, green arrow) and an obliteration of EPP depression throughout sustained stimulation (Figure 4D, blue arrow). Taken together, these data indicate that Lynx1 modifies the biophysical properties of the muscles and can influence synaptic properties.

### Loss of Lynx1 Alters the Contractile Properties of Skeletal Muscles

We used a force transducer to monitor muscle contractions induced by direct stimulation to assess the contractile properties of EDL muscles in Lynx1^−/−^ and WT control adult mice. We found that muscles lacking Lynx1 were less resistant to fatigue as compared to controls (control = 59.16% ± 2.24%; Lynx1^−/−^ = 47.15% ± 3.83%, *p* = 0.0186 at 1 min; Figure 4E). These data further support roles for Lynx1 in modulating the biophysical characteristics of skeletal muscles.

### Lynx1 Plays a Minor Role in Sculpting Developing Neuromuscular Junctions

**FIGURE 5:**
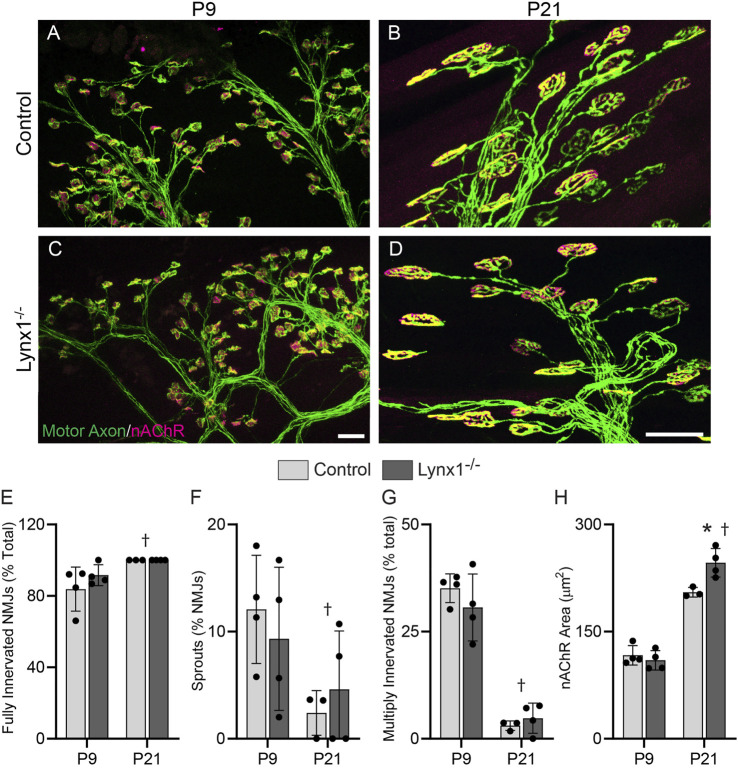
Loss of Lynx1 has no discernable impact on NMJ development. **(A–D)** Representative images of NMJs in the extensor digitorum longus (EDL) muscles of P9 **(A,C)** and P21 **(B,D)** control and Lynx1^−/−^ mice. Motor axons were labeled with YFP (green) and nicotinic acetylcholine receptors (nAChRs) were labeled with fluorescently conjugated α-bungarotoxin (fBTX, magenta). **(E–H)** Morphological analysis of neuromuscular junctions (NMJs), including **(E)** the degree of NMJ innervation, **(F)** the percentage of NMJs with axonal sprouts, **(G)** the percentage of NMJs innervated by more than one axon, and **(H)** NMJ area, as determined by the area of nAChR clusters. All values are mean ± SD. ^†^Age effect, *p* < 0.05, two-way ANOVA. *p < 0.05 versus age-matched control, two-way ANOVA with Šídák’s multiple comparisons test. *n* ≥ 3. Scale bar = 20 µm.

The expression pattern, interaction with NMJ-associated nAChRs, and the impact on the cholinergic transmission and contractile properties of skeletal muscles (Figures 2–4) suggested important roles for Lynx1 in the development and maintenance of NMJs. To begin to address this possibility, we examined NMJs in the EDL muscle of P9 and P21 control and Lynx1^−/−^ mice. Between these ages, NMJs and muscle fibers undergo fast and dramatic morphological, molecular, and functional changes (Sanes and Lichtman, 1999). Loss of Lynx1 had no impact on P9 NMJs (Figure 5) and only moderately caused the postsynapse to increase in size at P21 (Figure 5). Hence, Lynx1 is dispensable for the normal development of NMJs.

### Lynx1 Deletion Increases the Incidence of Age-Related Changes at Adult Neuromuscular Junctions

**FIGURE 6:**
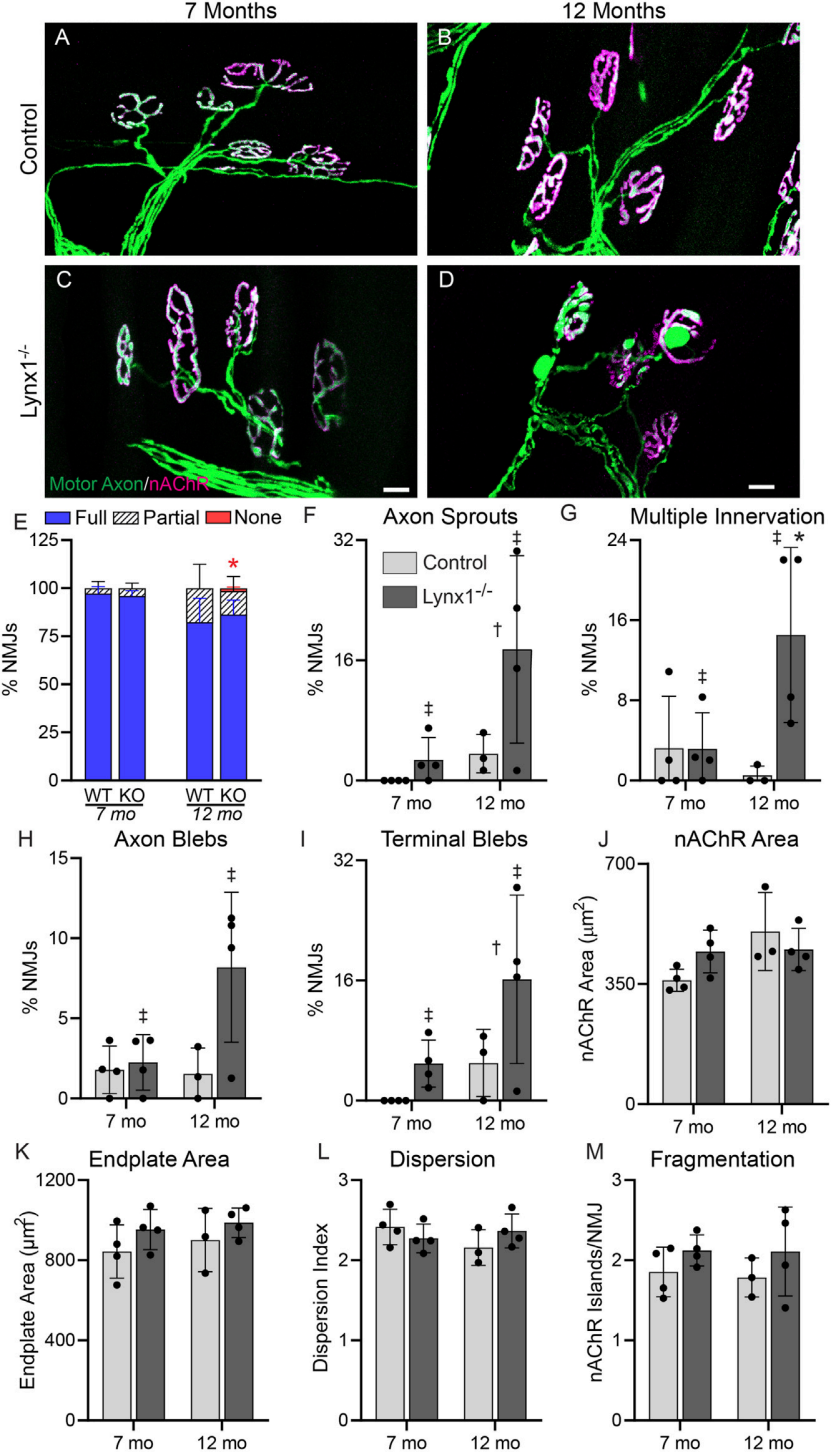
Lynx1 deletion increases the incidence of age-related changes at adult NMJs. **(A,B)** Representative images of neuromuscular junctions (NMJs) in the extensor digitorum longus (EDL) of 7-month-old **(A,C)** and 12-month-old **(B,D)** control and Lynx1^−/−^ mice. Motor axons were labeled with YFP (green), and nicotinic acetylcholine receptors (nAChRs) were labeled with fluorescently conjugated α-bungarotoxin (fBTX, magenta). **(E)** The percentage of innervated NMJs, either fully, partially, or not at all. **(F–M)** Morphological analysis of NMJs, including **(F)** the presence of axon sprouts, **(G)** multiple innervations, **(H)** the presence of preterminal axon blebs, **(I)** the presence of blebs in the axon terminal, **(J)** nAChR area, **(K)** endplate area, **(L)** nAChR cluster dispersion, and **(M)** nAChR fragmentation. *n* = 3–4. *p < 0.05 versus age-matched control; ^†^age effect, *p* < 0.05; ^‡^genotype effect, *p* < 0.05; two-way ANOVA with Šídák’s multiple comparisons test. All values are mean ± SD. Scale bar = 20 µm.

We next examined adult (7 and 12 months old) control and Lynx1^−/−^ mice. First, we assessed the physical activity of these mice by monitoring the distance traveled in running wheels inside their cages (Supplementary Figure S1). At 7 months of age, we found no difference in the distance traveled between genotypes. However, 12-month-old mice lacking Lynx1 did not travel as far as age- and sex-matched control mice. Next, we inspected NMJs for morphological changes associated with aging and diseases. This analysis showed no difference in the size of the NMJ between age- and sex-matched control and Lynx1^−/−^ mice (Figure 6A–D, J). However, it revealed a stark increase in the number of NMJs exhibiting axon terminal blebs in apposition to postsynaptic regions devoid of nAChRs (Figure 6D, I), nerve sprouts (Figure 6F), and large blebs in preterminal axons (Figure 6H) in Lynx1^−/−^ mice at both 7 and 12 months compared to control mice. The incidence of NMJs innervated by more than one motor axon was also higher in 12-month-old Lynx1^−/−^ compared to control mice (Figure 6G). The sustained and increased incidence of NMJs with deleterious structural changes between 7- and 12-month-old Lynx1 null mice suggested important roles for Lynx1 in maintaining NMJs as mice aged. Supporting this possibility, we found a trend towards altered expression of NMJ-associated genes specifically in 12-month-old Lynx1 null muscles (Supplementary Figure S2). For instance, both the alpha and epsilon nAChR subunits are expressed at lower levels in Lynx1 null compared to control muscle.

### Muscle Fibers are Smaller but Do Not Exhibit Signs of Degeneration in Lynx1^−/−^ Mice

**FIGURE 7:**
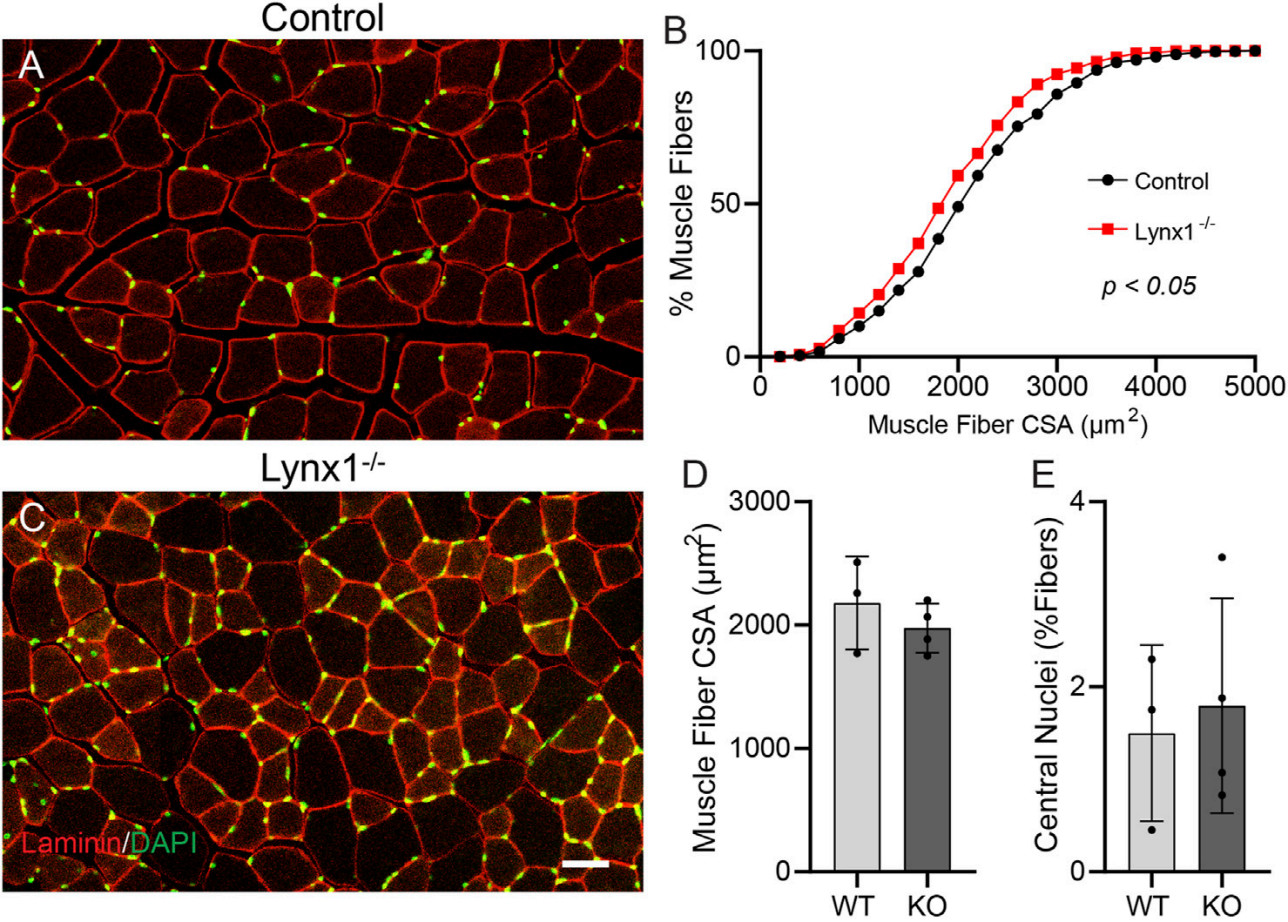
Muscle fibers are smaller but do not exhibit signs of degeneration in Lynx1^−/−^ mice. **(A,C)** Representative images of tibialis anterior (TA) cross sections from 12-month-old control and Lynx1^−/−^ mice in which muscle fibers are identified by laminin (red) and nuclei are labeled with DAPI (green). **(B)** Cumulative frequency of muscle fiber cross-sectional area (CSA). *p* < 0.05, Kolmogorov–Smirnov test. **(D)** The average muscle fiber CSA. **(E)** The percentage of muscle fibers with a centrally located nucleus. All values are mean ± SD. Scale bar = 50 µm.

We next asked whether loss of Lynx1 adversely affects adult muscle fibers. We examined muscle fibers for signs of atrophy, including reduced muscle fiber size and increased presence of muscle fibers undergoing cycles of degeneration and regeneration. Specifically, we measured the cross-sectional area (CSA) of skeletal muscle fibers in the TA muscle. We also determined the incidence of regenerating muscle fibers based on the presence of myonuclei near the center of the sarcoplasm (Folker and Baylies, 2013). A frequency histogram and a two-sample Kolmogorov–Smirnov (KS) test revealed that the TA muscle of 12-month-old Lynx1^−/−^ mice is populated by significantly smaller muscle fibers (Figure 7B). However, the average CSA of individual muscle fibers is not statistically different between mice with and without Lynx1 at 12 months of age (Figure 7D). We also found no difference in the number of muscle fibers with centralized nuclei between genotypes (Figure 7E).

### Loss of Lynx1 Does Not Affect the Rate of Neuromuscular Junction Reinnervation Following Denervation

**FIGURE 8:**
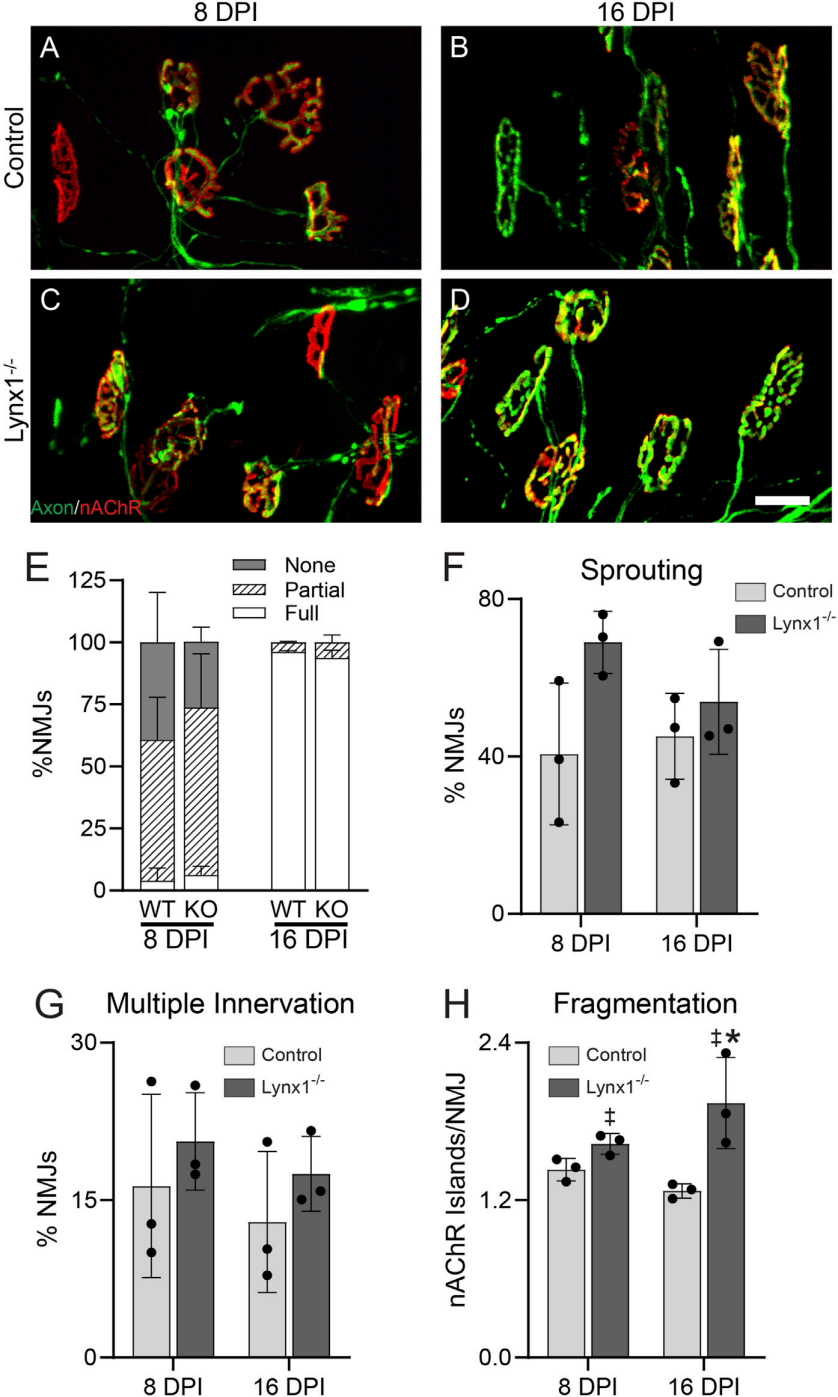
Loss of Lynx1 does not affect the stability nor repair of neuromuscular junctions (NMJs) following denervation. **(A,B)** Representative images of NMJs in the extensor digitorum longus (EDL) at 8 **(A,C)** and 16 days post-nerve crush injury (DPI). Motor axons were labeled with YFP (green), and nicotinic acetylcholine receptors (nAChRs) were labeled with fluorescently conjugated α-bungarotoxin (fBTX, red). **(E)** The percentage of innervated NMJs, either fully, partially, or not at all. **(F–H)** Morphological analysis of NMJs, including **(F)** the presence of axon sprouts, **(G)** multiple innervations, and **(H)** nAChR fragmentation. *p < 0.05 versus injury-matched control; ^‡^genotype effect, *p* < 0.05; two-way ANOVA with Šídák’s multiple comparisons test. *n* = 3. All values are mean ± SD. Scale bar = 25 µm.

The data above, together with Lynx1 regulation by two nerve-derived factors, ACh and z-agrin, suggested that Lynx1 may coordinate post- and pre-synaptic NMJ remodeling following denervation. To explore this possibility, we examined the morphology of NMJs in the EDL at 8 and 16 days after crushing the fibular nerve in Lynx1^−/−^;Thy1-YFP mice and age- and sex-matched control Thy1-YFP mice (Figure 8A–D). An analysis of the innervation status of NMJs, demarcated by YFP-labeled motor axons and fluorescently conjugated alpha-bungarotoxin (fBTX)-labeled nAChRs, revealed a similar rate of NMJ reinnervation at 8 days post-injury between Lynx1^−/−^ and control mice (Figure 8E). By 16 days post-injury, the majority of NMJs are fully innervated in both genotypes (Figure 8E–H). These data show that Lynx1 is not important for the structural repair of damaged adult NMJs. It is worth noting, however, that we did not assess the functional characteristics of previously denervated NMJs nor muscle fibers lacking Lynx1.

## Discussion

This study provides critical insights about Lynx1 in developing, adult, and denervated skeletal muscles and NMJs. We show that Lynx1 expression increases during development and is influenced by functional and structural changes at NMJs. We then demonstrate that Lynx1 binds nAChRs and affects muscles’ response to cholinergic transmission and contractile properties. We also show that while Lynx1 is largely dispensable for the development and repair of damaged adult NMJs, it plays important roles in NMJ maintenance. Thus, Lynx1 is an important member of the cholinergic system in the postsynaptic region of the NMJ.

### Expression and Distribution of Lynx1 in Muscles

We show that Lynx1 levels track structural and functional changes at NMJs. Lynx1 increases during development and concentrates at NMJs in the TA and EDL muscles. We also found that Lynx1 expression is regulated by two nerve-derived signals, ACh and z-agrin, critical for the function and stability of the NMJ. However, Lynx1 sharply decreases between 1 and 7 days post-injury, a period when muscles are completely denervated or in the early stages of NMJ reinnervation. In contrast to the period of denervation and early reinnervation, we observed the upregulation of Lynx1 expression at 9 and 12 days post-injury, a period of synaptic refinement and maturation. This expression pattern mirrors that reported in the brain (Morishita et al., 2010; Nabel and Morishita, 2013; Takesian et al., 2018) and suggests roles for Lynx1 in the development and repair of NMJs. In the developing CNS, Lynx1 upregulation drives the closure of enhanced synaptic plasticity of cholinergic circuits (Morishita et al., 2010; Sajo et al., 2016). However, we found no morphological differences between NMJs of Lynx1^−/−^ and control mice at P9 or P21. We also found that loss of Lynx1 has no effect on the rate of NMJ reinnervation and refinement following severing of motor axons. These data show that skeletal muscles do not use Lynx1 to promote the formation and maturation of NMJs. The possibility that Lynx1 impacts nAChR function without affecting NMJ morphology during development and following nerve injury, however, was not explored in this study.

### Lynx1 Interaction With Muscle–nAChR Subunits

Previous studies have uncovered two mechanisms by which Lynx1 interacts with CNS-specific nAChRs, including at the interface of α4/α4 nAChR subunits in the endoplasmic reticulum (Nichols et al., 2014) and at the extracellular domain of nAChR pentamers at the cell surface (Lyukmanova et al., 2013). In pull-down assays, we show that Lynx1 interacts with muscle-specific nAChR pentamers in HEK293 cells co-transfected with Lynx1 and the α1, β1, δ, and ε nAChR subunits. Interestingly, we were able to efficiently pull down α1 nAChR–Lynx1 complexes with purified BTX. This result suggests that Lynx1 either binds to a different region in the extracellular domain of nAChRs or its competition with BTX for binding to nAChRs is limited.

### Roles of Lynx1 at the NMJ and in Muscles

Our electrophysiological analysis shows faster MEPP rise time and the unraveling of potentiation followed by an absence of EPP depression elicited by sustained motor nerve stimulation in adult skeletal muscles lacking Lynx1. These findings are consistent with Lynx1 playing an important role in modulating the function of muscle nAChRs, similar to its role in the CNS (Miwa et al., 1999; Ibanez-Tallon et al., 2002). Therefore, it is possible that skeletal muscles also utilize Lynx1 to modulate nAChRs to preserve synaptic health by controlling the timing of homeostatic plasticity. While the NMJ remains relatively stable throughout adulthood, it experiences intermittent homeostatic plasticity events resulting from the “wear and tear” of growing older. While such bouts of plasticity in adulthood likely support NMJ adaption to changes in cholinergic signaling, they must close in a timely fashion for NMJs to transduce cholinergic signaling within the physiological range for muscles to remain viable. Supporting this function for Lynx1, its deletion increased the incidence of NMJs exhibiting deleterious structural changes in 7- and 12-month-old mice. The loss of Lynx1 also altered muscle fatigue resistance and reduced the size of muscles fibers. In stark contrast, Lynx1 deletion had no impact on the development nor repair of NMJs. These data strongly suggest that Lynx1 plays important roles in NMJ maintenance but not NMJ development or repair.

### Future Studies on Lynx1 at Neuromuscular Junctions and in Skeletal Muscles

While this study unravels a number of key roles of Lynx1 at NMJs and in skeletal muscles, it also raises a number of additional questions. Of particular interest is the identity of molecular mechanisms downstream of ACh and z-agrin that regulate Lynx1 expression in skeletal muscles. Lynx1regulation by other signaling pathways associated with muscle fiber maturation (Venuti et al., 1995) and NMJ stabilization as well as post-translational modifications of Lynx1 by GPI-cleaving enzymes (Liu and Fujita, 2020) cannot be ruled out. A better understanding of how pathways located both up- and downstream of Lynx1 are affected by synaptic changes will provide a broader understanding of the role of Lynx1 at the NMJ. A second outstanding question is the mechanistic link between postsynaptic Lynx1 and presynaptic integrity. This is of particular interest since loss of Lynx1 causes several presynaptic abnormalities in adult and middle-aged mice. It is possible that muscle-derived Lynx1 could impact presynaptic integrity through its influence on nAChR stability, a possibility not explored in this study. Through its impact on nAChRs, Lynx1 may influence the expression of muscle-derived molecules such as FGFBP1 (Taetzsch et al., 2017), Dach2, and HDAC9 (Macpherson et al., 2015) and, in turn, impact the stability, function, and health of the presynapse. A better understanding of these unanswered questions about Lynx1 could shed light on opportunities to preserve the structure and function of NMJs and muscles during aging and other chronic conditions, including amyotrophic lateral sclerosis (ALS).

## Data Availability

The original contributions presented in the study are included in the article/Supplementary Materials, further inquiries can be directed to the corresponding author.
