# Supplementary figures and images for: Expression and Roles of Lynx1, a Modulator of Cholinergic Transmission, in Skeletal Muscles and Neuromuscular Junctions in Mice
Source: Front Cell Dev Biol. 2022 Mar 16;10:838612. doi: 10.3389/fcell.2022.838612 (PMC8967655; doi:10.3389/fcell.2022.838612)

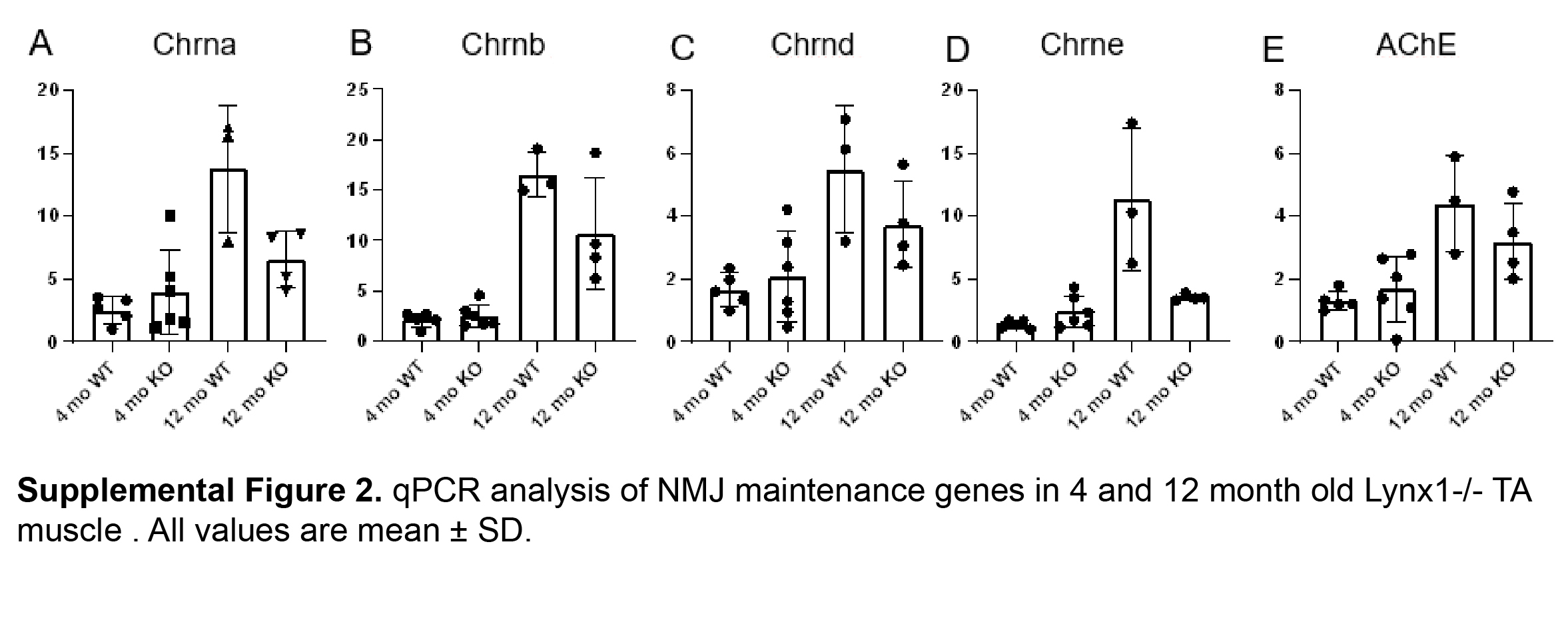

Supplement: Supplementary file 1 [file Image1.jpeg]

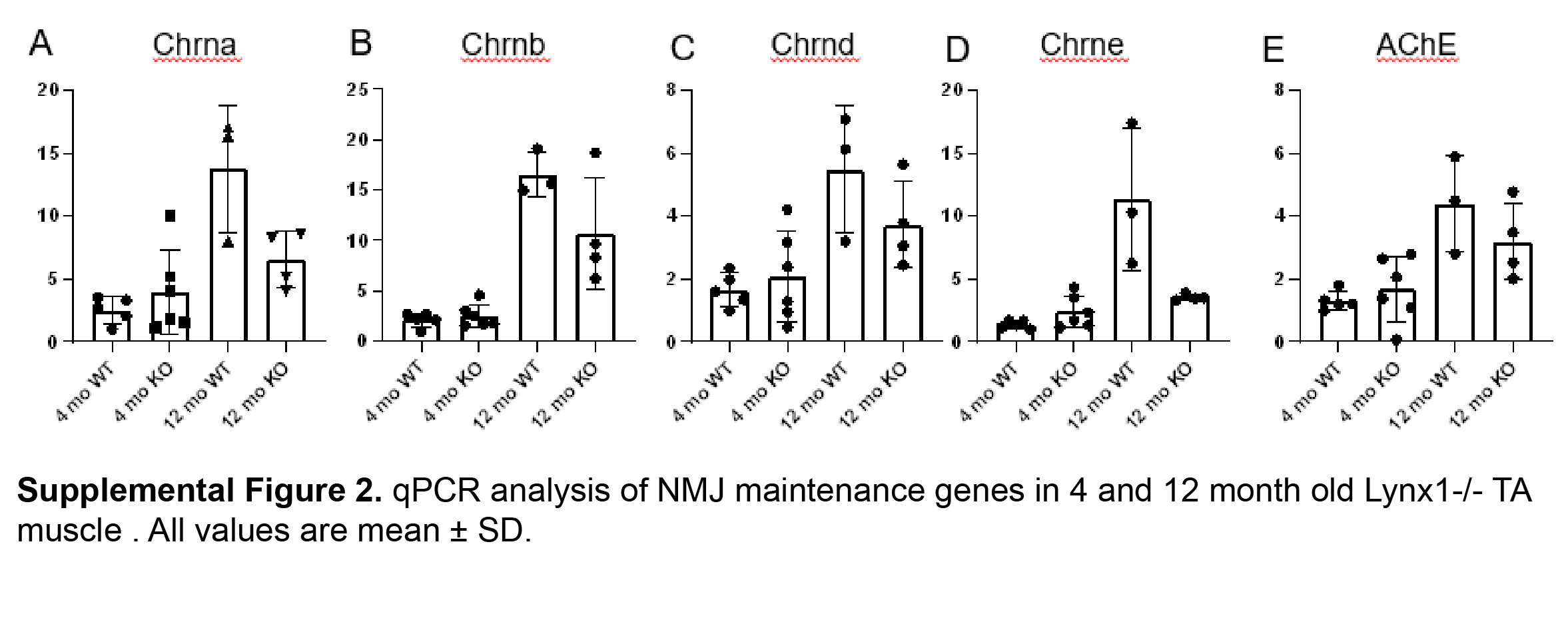

Supplement: Supplementary file 2 [file Image2.JPEG]
